# Healthy Eating and Active Living for Diabetes-Glycemic Index (HEALD-GI): Protocol for a Pragmatic Randomized Controlled Trial

**DOI:** 10.2196/11707

**Published:** 2019-03-06

**Authors:** Hayford M Avedzi, Kate Storey, Jeffrey A Johnson, Steven T Johnson

**Affiliations:** 1 School of Public Health University of Alberta Edmonton, AB Canada; 2 Centre for Nursing and Health Studies Faculty of Health Disciplines Athabasca University Athabasca, AB Canada

**Keywords:** glycemic index, randomized controlled trial, type 2 diabetes mellitus

## Abstract

**Background:**

Rigorous evidence is needed regarding the best approach for increasing the uptake of Diabetes Canada’s evidence-based recommendations to include low-glycemic index (GI) foods in daily meal planning as an effective dietary self-care strategy for glycemic control among people with type 2 diabetes (T2D).

**Objective:**

This study aims to present the study design and baseline data from the Healthy Eating and Active Living for Diabetes-Glycemic Index (HEALD-GI) trial, which was designed to evaluate the effectiveness of an enhanced GI-targeted nutrition education on GI-related knowledge and mean daily GI among adults with T2D in Edmonton, Alberta.

**Methods:**

We used a pragmatic randomized controlled trial design and allocated 67 adults (aged ≥18 years) with T2D living in Edmonton, Alberta, Canada, to a control group that received standard printed copies of Canada’s Food Guide and Diabetes Canada’s GI resources or to an intervention group that received the same materials, plus a customized Web-based platform with 6 self-directed learning modules and print material. Each module included videos, links to reliable websites, chat rooms, and quizzes. Evidence-based GI concept information included GI values of foods and low-GI shopping, recipes, and cooking tips by a registered dietitian. In addition, support through email, text messaging (short message service), phone calls, or postal mail was provided to reinforce participants’ learning. The primary outcome, average dietary GI, was assessed using 3-day food records. Additional measures including GI knowledge and self-efficacy, glycated hemoglobin (HbA_1c_), lipids, systolic blood pressure, body mass index (BMI; weight, height), waist circumference, and computer proficiency were assessed at baseline and at 3-month postintervention.

**Results:**

Between November 2017 and February 2018, we contacted adults (aged ≥18 years) with T2D living in Edmonton, Alberta, screened and recruited eligible participants into the study. All data collection ended in June 2018. Overall, 64% (43/67) participants were males; mean age was 69.5 (SD 9.3) years, with a mean diabetes duration of 19.0 (SD 13.7) years. Mean BMI was 30.1 (SD 5.7) kg/m^2^, and mean HbA_1c_ value was 7.1% (SD 1.2%). Data analysis was completed in December 2018.

**Conclusions:**

The GI concept is often difficult to teach. The HEALD-GI study aims to provide evidence in support of an alternative approach to translating the GI concept to adults with T2D. Findings from this study may help registered dietitians to better disseminate low-GI dietary recommendations using efficient and cost-effective, patient-centered approaches. Furthermore, evidence generated will contribute to addressing some of the controversies regarding the clinical usefulness of the GI concept.

**International Registered Report Identifier (IRRID):**

DERR1-10.2196/11707

## Introduction

Increasing prevalence of type 2 diabetes (T2D) remains a major public health challenge with adverse effects for individuals and health care systems globally. Currently, diabetes affects approximately 7.3% of Canadians, and the prevalence has been projected to rise to about 10% by 2020 [1]. Health outcomes for individuals with diabetes, however, largely depend on their ability to self-manage the disease. Lifestyle interventions including healthy eating, physical activity, and smoking cessation, which enhance the acquisition of knowledge, skills, resources, and support to boost self-efficacy for day-to-day living, are, therefore, important for T2D management and prevention of long-term complications [2,3].

Healthy eating remains a key strategy for diabetes self-management, and dietary carbohydrates constitute one aspect of the diet with significant influence on blood glucose control. Different types and quantities of carbohydrates have been shown to impact blood glucose concentration differently [4,5]. This property of foods, referred to as glycemic index (GI), is used to rank how quickly a given dietary carbohydrate raises blood glucose concentration immediately following a meal [4]. Using GI and the portion size of a given food, glycemic load (GL), a composite measure of carbohydrate quality and quantity, can be calculated to predict blood glucose response to a specific type and amount of a dietary carbohydrate [6]. Consuming low-GI foods is beneficial for metabolic control in diabetes management [7]. Adopting a low-GI dietary pattern as part of a healthy eating lifestyle has been shown to markedly reduce cardiovascular risk factors (eg, total cholesterol, high-density lipoprotein); improve glycemic control, postprandial glycemia, and beta cell function; and decrease the need for antihyperglycemic agents among patients with diabetes [8-18]. Outlining effective approaches to promoting the concept of low-GI intake among individuals with T2D has been problematic. Hence, examining effective modes of delivery is necessary to support one aspect of T2D.

Effective and widespread use of information technologies (ITs), including the internet and mobile-based tools, is revolutionizing the traditional approaches for engaging, educating, and empowering individuals with chronic diseases such as diabetes [19,20]. Increasing use of IT by diverse audiences, occasioned by low-cost Web- and mobile-based tools may, therefore, offer viable prospects for promoting swift and cost-effective GI concept education and support among people with T2D. Features of modern IT tools such as websites, chat rooms, social networking sites (eg, Facebook), and short message service (SMS) text messaging apps allow creation and exchange of health-promoting information and enable individuals to interact with other users who share connections with them [21,22]. Properly designed and managed websites can serve as credible sources of evidence-based GI-targeted messages. Websites enable integration and presentation of text, graphics, or audiovisuals on one platform, while chat rooms and emails facilitate engagement between users and health professionals in addressing pertinent issues [23]. Furthermore, chat rooms provide Web-based social forums for peer group discussions, exchange of ideas, encouragement, and support [24,25].

Knowledge gaps exist between GI concept clinical guidelines and their translation to adults with T2D in Canada due to debates over clinical utility of GI, inconsistencies in teaching the GI concept by registered dietitians [26,27], and limited patient-dietitian interactions [28]; these limit patients’ knowledge and skills for uptake of GI dietary behavior. Patients lose out on the additional benefits of improving carbohydrate quality by consuming healthy, low-GI foods as part of healthy eating strategy for diabetes self-management. Hence, this study aims to examine the effectiveness of Web-based, GI-targeted nutrition education on dietary behavior and intakes among adults with T2D. We hypothesize that after 3 months, adults with T2D who were randomized to receive Web-based, GI-targeted nutrition education will consume a lower-GI diet and show improved glycemic control compared with a control group. Findings from this study will help determine whether, and how, current approaches to disseminating dietary recommendations pertaining to GI concept could be improved for better uptake using alternative, efficient, and cost-effective patient-centered approaches to nutrition self-care. Furthermore, the outcomes of this study will add to the body of evidence regarding the GI concept.

## Methods

### Study Design: Setting, Recruitment, Ethical Considerations, and Intervention

#### Setting and Population

Adults (aged ≥18 years) living in Edmonton, Alberta, Canada, with T2D and currently enrolled in the Alberta’s Caring for Diabetes (ABCD) cohort study [29] constituted the target population for this study. The ABCD cohort study [29] was designed to explore different aspects of diabetes care and the development of complications among individuals with T2D in Alberta. The ABCD cohort enrolled 2040 participants between 2012 and 2013 from all over the province of Alberta at inception and provided a suitable eligible population from which to draw participants for this intervention. The characteristics of the ABCD cohort have been reported elsewhere [29].

#### Inclusion and Exclusion Criteria

The inclusion criteria were as follows: individuals aged ≥18 years identified as having T2D and currently enrolled in the ABCD cohort study; able to read, understand, and converse in English; and willing to provide informed consent. For practical considerations, we prescreened all cohort participants living in Edmonton for enrollment in the intervention. Based on postal codes, 745 cohort participants who participated in the year 1 ABCD survey lived in Edmonton. However, due to relocation and mortality-related attrition, we invited only 485 ABCD cohort participants living in Edmonton between July 2017 and October 2017 to participate. Those who responded were screened for eligibility and subsequent recruitment into the study between November 2017 and February 2018. Those taking exogenous insulin and having physiological or medical conditions that interfere with usual digestive functions were excluded.

#### Participants Screening Procedures

We sent letters explaining details of the study to all eligible participants, asking them to contact the study staff if they were interested in participating in this study. We performed detailed prescreening over the phone to determine full eligibility once we received responses from those invited to participate. A maximum of 2 telephone contacts were made to remind eligible individuals who did not contact the research staff after 2 weeks of expressing their interest in participating in the study. We invited eligible participants to complete baseline anthropometry, biochemical, clinical, and dietary data collection at the Human Nutrition Research Unit located within the Alberta Diabetes Institute at the University of Alberta. A trained dietitian and registered nurse with data collection experience collected anthropometric data and relevant clinical measures using point-of-care instruments (DCA Vantage, Cholestech LDX, and BPTru).

#### Ethical Considerations

The University of Alberta and Athabasca University Health Research Ethics Boards reviewed and approved the study protocol. In line with research ethics requirements, all participants received adequate information about the study and had the opportunity to ask questions. We obtained written informed consent from participants prior to obtaining any study measurements after providing them an explanation of (1) the purpose of the study; (2) the allocation process; (3) the use of data and the means of assuring confidentiality; (4) voluntary participation and the participants’ right to withdraw from the study at any time; and (5) any potential harm that could occur as a result of the intervention.

### Randomization and Treatment Allocation

Using a pragmatic randomized trial design, we randomly allocated 67 eligible participants drawn from the ABCD cohort [29] who provided informed consent and completed baseline anthropometry, clinical, and dietary measurements to either the usual care or to intervention in a 1:1 ratio using a computer-generated allocation sequence (Stata SE 12.1; StataCorp) [30]. Allocation sequence and group assignments were generated centrally and enclosed in sequentially numbered and sealed envelopes. A statistician not involved with other aspects of the trial performed all randomization-related procedures.

#### Usual Care

Study participants allocated to the usual care (control arm) received standard printed copies of Canada’s Food Guide and Diabetes Canada (formerly Canadian Diabetes Association) GI resources in line with current Diabetes Canada Clinical Practice Guidelines [31,32]. The control group did not receive extra support aimed at increasing knowledge or skills for daily consumption of low-GI foods.

#### Enhanced Glycemic Index-Targeted Nutrition Education

Participants (n=33) allocated to the enhanced low-GI education intervention group received the same information as the control group in addition to vetted, evidence-based, learner-centered, low-cost, and actionable low-GI messages delivered through a Web-based platform with chat rooms, customized videos featuring a registered dietitian, and print material. Based on individual preference and needs, we provided additional support through email, SMS text messages, phone calls, or mail to reinforce participants’ learning. The GI concept and content of this intervention were in line with current Diabetes Canada Clinical Practice Guidelines as well as T2D patients’ suggested content and preferred modes of learning GI information [16,25]. The intervention website was managed by a trained research assistant who also moderated chat room discussions under the supervision of coinvestigators: KS, a registered dietitian and researcher, and STJ and JAJ, who also possess extensive research experience.

Participants received brief tutorials on website log-in, navigation, and usage during baseline data collection, which was reinforced at first log-in with a short video introduction to the program. The video emphasized the importance of low-GI eating and summarized the various aspects of the intervention. Those allocated to the intervention group covered a total of 6 modules over 12 weeks. These modules were aimed at enhancing knowledge and skills for improved GI dietary behavior change. Each module included customized videos featuring a registered dietitian, links to reliable websites, chat rooms for social support, and quizzes. Evidence-based GI content included GI values of foods; low-GI shopping, recipes, and cooking tips; and advice for eating out. Participants received a new module every 2 weeks. Specific topics covered in these modules included (1) general healthy eating for diabetes patients; (2) summary of the GI concept; (3) identifying, choosing, and shopping low-GI foods; (4) low-GI recipes, menus, and meal planning; (5) guidelines for eating out and snacking; and (6) GI concept and general diabetes self-management and healthy lifestyles. Participants were encouraged to outline and track personal, easily achievable goals that could enhance their GI knowledge and skills under each module. For example, in module 3, a participant could set a simple goal to learn how to identify low-GI versions of foods that he or she usually consumed. We delivered each module through the intervention website using user-friendly text, graphical displays, and module summary videos. All videos were developed in line with Canada’s Food Guide and Diabetes Canada Nutrition Therapy Clinical Practice Guidelines-based GI recommendations [31,32] to teach participants “hands-on” application of GI to daily meal planning.

To sustain enthusiasm, participants were granted access to subsequent modules at the end of the preceding module on the first day of each 2-week cycle; this was accompanied with electronic reminders delivered through email or SMS text message based on participants’ choosing. Participants responded to short quizzes meant to bolster key GI principles and lessons learned. Chat rooms were activated for each module to enable participants to share experiences in the form of success stories, challenges, and tips that enhance a sense of community for social support among participants. The chat room forum was monitored, and timely responses were provided to questions and concerns of participants. In addition, we provided weblinks to the international tables of GI and GL values of foods [33] and additional evidence-based information on GI concept as well as general diabetes self-management and healthy lifestyles. A review of similar Web-based studies has shown that interventions that provide interactive elements such as the following have been effective at generating and sustaining participants’ interest and exposure to Web-based interventions: (1) quizzes, searchable database, audio or video; (2) counselor support through counselor-led chat sessions, email, or phone contacts; (3) peer support through Web-based discussion forums or chats; and (4) regular updates of information on intervention websites [34].

Participants in the enhanced GI education arm also received copies of “The Shopper’s Guide to GI Values: the Authoritative Source of Glycemic Index Values for More Than 1,200 Foods” [35] in line with T2D patients’ preference for print-based material as a source GI information [25]. Briefly, the Shopper’s Guide, which was recommended to participants seeking to know more about GI in a previous study [36], is a lightweight, handy book coauthored by expert GI research scientists. It contains GI values of over 1200 foods arranged by categories to help identify healthier low-GI carbohydrate alternatives using handy household measures. The Shopper’s Guide is updated regularly and has comprehensive data on carbohydrates per serving and GL, a shopping list of low-GI essentials, ideas for gluten-free meals, facts about sugar and sweeteners, and tips for everyday meals and dining out. Furthermore, the Shopper’s Guide provides links to supplementary resources with reliable, evidence-based GI information [35].

In addition to the website and the Shoppers Guide [35], participants in the intervention arm were offered periodic emails, SMS text messages or telephone calls, or postal mail prompts to visit the website and/or use the print materials to acquire more GI knowledge as per individual preference. Participants were encouraged to use these mediums to seek assistance regarding specific personal dietary issues, which they may not want to post in the chat room discussion section of the website.

### Assessment of Study Outcomes

#### Primary Outcome

Our primary outcome measure was GI-related dietary behavior change and intake, measured using a 3-day food record.

#### Dietary Assessment, Glycemic Index, and Glycemic Load Estimation

Daily dietary intake was assessed for all participants at baseline and at 3 months using a 3-day food record. The 3-day food records are valid and reliable for capturing dietary behavior change by asking participants to record their food consumption as they eat [37]. All participants were asked to record, in as much detail as possible, descriptions of foods and beverages consumed over a 3-day period (ie, 2 weekdays and 1 weekend day). Participants were given further instructions on how to fill out the 3-day food record. Color photographs were provided to assist participants with estimating and recording appropriate portion sizes of foods and beverages they consumed in the 3-day food record logbooks. Pictures showing sample portions sizes of foods measured against items including a finger, palm of a hand, and a hockey puck were included and participants were encouraged to choose photographs that best represented their portion sizes or specify whether they consumed more or less. Mean daily food consumption and nutrient intake were estimated using the Food Processor Diet Analysis and Fitness Software (ESHA Research) at baseline and 3 months using the Canadian nutrient file.

**Figure 1:**
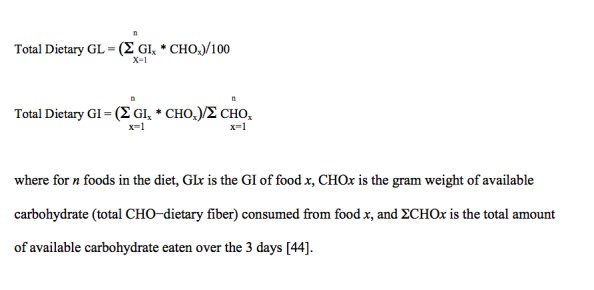
Mean daily glycemic index (GI) and glycemic load (GL) calculation.

All carbohydrate-containing foods identified from the 3-day food records were assigned GI values corresponding to the best geographic and botanical matches in the published International Table of Glycemic Index and Glycemic Load Values [33,38] or the updated University of Sydney Web-based database [39,40]. GI values were averaged for foods having more than one GI value from very similar matches. As the International Table and the University of Sydney Web-based databases do not provide an exhaustive entry of glycemic data for every food, the instances where foods could not be matched directly to those in the International Tables or Web-based database, GI values were calculated from the estimated GL [39] or matched to listed foods with similar characteristics (ingredients, composition, and physical properties) based on all information available to HMA, a trained dietitian, and from his subjective experience and knowledge of foods [41-43]. As recommended [41,43,44], daily average GI and GL was calculated for all carbohydrate-containing foods identified from the 3-day food records using published international GI tables [33,38] (Figure 1).

#### Secondary Outcomes

Secondary outcome measures, including GI knowledge and skills, self-efficacy, body mass index (BMI; weight and height), waist circumference, clinical measures (glycated hemoglobin [HbA_1c_], systolic blood pressure, total cholesterol, and high-density lipoprotein), and computer proficiency were assessed at the baseline and at 3 months after completing the intervention. In addition, demographic data were collected.

#### Glycemic Index Knowledge and Self-Efficacy Assessment

Pre- and postintervention GI concept knowledge and self-efficacy were assessed and quantified using the Glycemic Index Foods Quiz from a previous study [45]. Dietary data from a previous intervention within the same population showed that, out of 196 participants, 16% were not familiar with low-GI eating and 28% did not include low-GI foods in their diets [46]. About 35% (70/199) indicated that they did not know about GI, and of those who claimed the knowledge of GI, only 34% reported choosing low-GI foods for >6 months in another study [47]. These corroborate previous findings in which only 38% of people with diabetes received nutrition therapy across Canada [28] with <40% of dietitians including the GI concept in T2D dietary self-care counseling [26,27]. Overall dietary self-care behavior was assessed using dietary items in the validated and widely used Summary of Diabetes Self-care Activities measure [48]. The Glycemic Index Foods Quiz, therefore, enabled assessment of the net change in GI knowledge and self-efficacy due to the intervention.

#### Clinical and Anthropometric Measures

Clinical outcome measures included HbA_1c_, systolic blood pressure, and lipid profile. Capillary blood samples (35 μL) were collected from participants to assess HbA_1c_ value using previously validated point-of-care testing device for HbA_1c_ (DCA Vantage) [49] and lipid profile (Cholestech LDX). Systolic blood pressure was measured according to the standard protocols using (BPTru) [50].

In addition, weight, height, and waist circumference were assessed according to the Canadian Physical Activity, Fitness and Lifestyle Appraisal procedures [51]. Body weight in kilograms (kg) and height in meters (m) were measured for each subject in light clothing and with no shoes on. Body weight was measured to the nearest 0.1 kg with a portable digital scale (Tanita BWB-800S, Arlington Heights, IL, USA), and height was measured using a portable stadiometer (Tanita HR-100). Waist circumference was measured to 1 mm at the top of the iliac crest using a spring-loaded Gulick anthropometric tape (FitSystems Inc, Calgary, AB, Canada). Regular, monthly quality assurance checks were conducted on the point-of-care devices and scale.

#### Physical Activity

Self-reported physical activity was assessed using the Godin Leisure Time Exercise Questionnaire [52]; the validity of this questionnaire is well established [53], and data suggest that self-reported physical activity estimates function as a suitable predictor of future behavior [54]. Participants were asked to report the frequency and duration of light-, moderate-, and vigorous-intensity leisure-time physical activity that lasted at least 10 minutes over a typical week during the past month. The number of weekly minutes for each intensity level was calculated by multiplying the frequency of activity by the duration in minutes. The sum of weekly minutes of moderate-to-vigorous physical activity (MVPA) gave the total MVPA minutes per week.

#### Computer Proficiency

Participants’ computer proficiency was measured using the Computer Proficiency Questionnaire (CPQ) at baseline and 3-month postintervention. The CPQ was developed for evaluating the competencies of seniors with regards to use of computers and associated applications such as the internet [55]. The CPQ assesses competence across 6 different subscales: computer basics, printing, communication, internet, scheduling software (calendar), and multimedia use (entertainment) for gauging an individual’s specific and overall computer proficiency.

#### Sociodemographic Covariates

Demographic information including age, sex, marital status, education, employment status, income, and personal history of cardiovascular disease risk factors (eg, smoking) and time since T2D diagnosis were collected at baseline using a questionnaire.

#### Intervention Preference and Website Usage Data

Preference and usefulness of the Web-based, print [35], email, SMS text messages or telephone call, and postal mail were assessed by asking participants how many times they visited the webpage, read and made references to Shopper’s Guide [35], and how much time they spent on the website or reading the book. In addition, participants were asked which medium they found most helpful and whether the information about the GI concept was informative and helped increase their knowledge and self-efficacy for consuming low-GI foods. Website data measurement programs were built into the website design to compile data points as connections occur with the target audience [56]. Regularly collecting, tracking, and using measurement data makes it possible to understand participants’ characteristics and helps keep the intervention appealing and relevant for achieving the greatest effect [56].

### Statistical Analysis, Power, and Sample Size Rationale

#### Statistical Analysis

Change in the mean daily GI of dietary carbohydrates from baseline to 3 months will be used as our primary effectiveness measure for improved low-GI knowledge and application. Descriptive statistics will be computed for all variables to determine the nature of the data and to test for normality assumptions. Changes in outcomes will be assessed using repeated-measures 2-way analysis of covariance. Potential sociodemographic and clinical factors associated with enhanced GI learning will be evaluated using generalized linear mixed-model analysis. Treatment condition, baseline scores, participant characteristics (eg, sex, education, and income), and computer proficiency that may be significantly related to outcomes will be controlled for. All data will be analyzed using Stata SE 12.1 (StataCorp).

#### Power and Sample Size Rationale

Based on previous studies regarding the efficacy of GI-based nutritional education and glycemic control [14,45] and meta-analysis of studies on low-GI diets and diabetes management [8], an estimated effect size of *d*=1 was set for this intervention. Previous data [46] suggest an SD of GI intake of 4-5 units; thus, an effect size *d*=1 could be achieved with an absolute mean difference of 5 units of GI intake. Given the estimated effect size, 42 participants (21 per arm) would be sufficient for detecting an absolute mean difference of 5 units on the GI intake scale between means with an error of alpha=.05 (2-sided) and beta=.1 (power 1−β=.90; Multimedia Appendix 1); this difference is considered to have significant health benefits from a previous study in which a change of 15 GI units yielded a corresponding HbA_1c_ change of −1.5% [45] and another study in which a change of 4.6 GI units yielded an HbA_1c_ change of −0.25 (95% CI −0.50 to −0.004) [14]. With an estimated attrition rate of 30% (based on the ABCD cohort year 2 participation rate), eligible participants were oversampled (N=67) during recruitment to account for possible loss to follow-up during randomization and intervention periods. This sample size was feasible in view of the dietary assessment (3-day food record) method used to assess food intakes and change in dietary GI, the cost of biochemical and clinical measurements, and the duration of the study.

## Results

### Summary of Progress to Date

Intervention milestones including ethics application, hiring and training of research assistants, and development and pilot-testing of intervention materials ran from July 2016 to October 2017. Data Analysis was completed December 2018.

### Recruitment or Enrollment Status and Timelines

**Figure 2:**
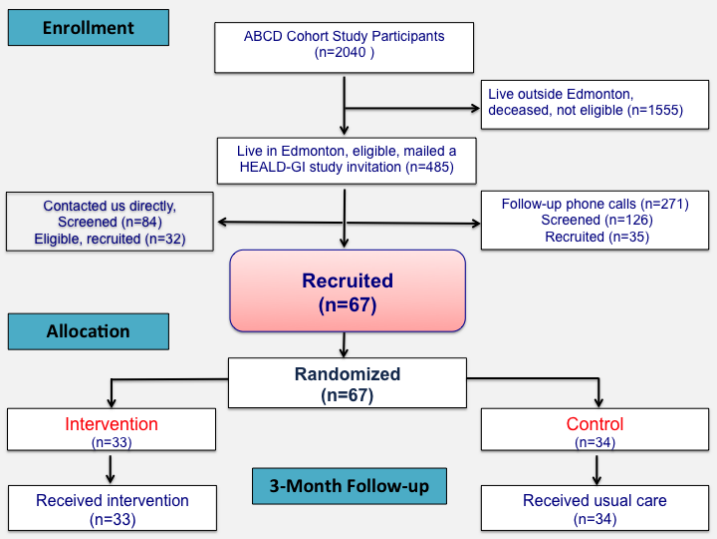
Flow diagram showing participant recruitment and treatment allocation in the Healthy Eating and Active Living for Diabetes-Glycemic Index (HEALD-GI) study.

Recruitment and enrollment in the Healthy Eating and Active Living for Diabetes-Glycemic Index (HEALD-GI) trial ran from November 2017 to February 2018. Figure 2 shows the flow diagram detailing the recruitment, screening, random allocation, and baseline and follow-up data collection. Baseline and 3-month follow-up data collection were completed in June 2018. Currently, the study database is being compiled in preparation for performing appropriate analyses and dissemination of findings.

### Participant Characteristics

Overall, 64% (43/67) participants were males; mean age was 69.5 (SD 9.3) years, with a mean diabetes duration of 19.0 (SD 13.7) years, BMI of 30.1 (SD 5.7) kg/m^2^, and HbA_1c_ value of 7.1% (SD 1.2%) (Table 1).

**Table 1:** Participant characteristics.

Characteristics		All (n=67)	Intervention (n=33)	Control (n=34)
Males, n (%)		43 (64)	20 (61)	23 (68)
Age (years), mean (SD)		69.5 (9.3)	70.7 (9.0)	68.4 (9.6)
**Marital status, n (%)**				
	Married or common law	47 (70)	20 (61)	27 (79)
	Not married (never married, widowed, divorced, or refused to answer)	20 (30)	13 (39)	7 (21)
**Ethnicity, n (%)**				
	Caucasian	62 (93)	30 (91)	32 (94)
	Non-Caucasian	5 (7)	3 (9)	2 (6)
**Education,** **n** **(%)**				
	High school and less	21 (31)	12 (9)	12 (35)
	College and higher	46 (69)	24 (73)	22 (65)
**Employment,** **n (%)**				
	Employed	10 (15)	2 (6)	8 (23)
	Unemployed	5 (7)	1 (3)	4 (12)
	Retired	52 (78)	30 (91)	22 (65)
**Annual household income (Can $), n (%)**				
	<40,000	8 (12)	3 (9)	5 (15)
	40,000-79,999	30 (45)	16 (49)	14 (41)
	≥80,000	20 (30)	7 (21)	13 (38)
	Do not know or refused to answer	9 (13)	7 (21)	2 (6)
Diabetes duration (years), mean (SD)		19.0 (13.7)	20.0 (11.7)	18.0 (15.5)
Glycated hemoglobin value (%), mean (SD)		7.1 (1.2)	7.0 (1.4)	7.1 (0.9)
**Lipid profile, mean (SD)**				
	Total cholesterol (TC; mmol/L)	4.4 (1.0)	4.3 (1.0)	4.5 (0.9)
	HDL (High-density lipoprotein; mmol/L)	1.3 (0.4)	1.4 (0.4)	1.3 (0.4)
	TC/HDL ratio	3.6 (1.5)	3.3 (0.9)	3.9 (1.9)
**Blood pressure (BP; mm Hg), mean (SD)**				
	Systolic BP	127.9 (12.4)	127.7 (9.9)	128.2 (14.6)
	Diastolic BP	70.1 (10.6)	69.8 (8.1)	70.5 (12.8)
Resting heart rate (bpm), mean (SD)		77.8 (14.5)	78.8 (15.3)	76.8 (13.9)
Body mass index (kg/m^2^), mean (SD)		30.1 (5.7)	28.0 (5.1)	32.0 (5.6)
Waist circumference (cm), mean (SD)		107.4 (16.1)	102.5 (15.5)	112.2 (15.4)

## Discussion

### Principal Findings

This protocol outlines the study rationale, design, and evaluation of the HEALD-GI pragmatic randomized controlled trial and reports the baseline characteristics of 67 individuals living with T2D in Edmonton, Alberta, Canada. The HEALD-GI trial was designed to evaluate the effectiveness of Web-based, GI-targeted nutrition education on GI-related knowledge and intakes among adults with T2D.

Major strengths of this trial include the evidence-informed components of the Web-based enhanced, GI-targeted nutrition education including internet chat rooms for peer support and use of email, SMS text messages, and telephone support, which have been shown to enhance the intervention uptake and effectiveness [34,57]. Emails, SMS text messages, and telephone support enable educational content-related exchanges, while chat room platforms in Web-based learning environments enhance social support through creation of relationships that support collaborative learning and sharing of relevant experiences [58-60]. In addition, the involvement of a health professional as a moderator of the Web environment has been shown to enhance Web-based intervention outcomes [57,61]. The provision of “The Shopper’s Guide to GI Values: the Authoritative Source of Glycemic Index Values for More Than 1,200 Foods” [35] also supports preferences for print-based material as a source GI information [25]. This may enhance participant knowledge and self-efficacy for low-GI concept uptake. Use of a 3-day food record method for dietary intake data will help curb recall bias, which is often associated with memory-dependent dietary assessment methods such as 24-hour recall [37].

### Conclusion

The GI concept is often difficult to teach. The HEALD-GI study aims to provide evidence in support of an alternative approach to translating the GI concept to adults with T2D. Findings from this study may help registered dietitians to better disseminate low-GI dietary recommendations using the efficient and cost-effective patient-centered approaches. Furthermore, evidence generated will contribute to addressing some of the controversies regarding debates surrounding the clinical usefulness of the GI concept.

## Appendix

Multimedia Appendix 1 Sample size and power calculations.

Multimedia Appendix 2 CFDR peer-review feedback.

## Abbreviations

ABCD: Alberta’s Caring for Diabetes Cohort Study
BMI: body mass index
CPQ: Computer Proficiency Questionnaire
GI: glycemic index
GL: glycemic load
HEALD-GI: Healthy Eating and Active Living for Diabetes-Glycemic Index
IT: information technology
MVPA: Moderate-To-Vigorous Physical Activity
SMS: short message service
T2D: type 2 diabetes
