# Supplementary material for: Healthy Eating and Active Living for Diabetes-Glycemic Index (HEALD-GI): Protocol for a Pragmatic Randomized Controlled Trial
Source: JMIR Res Protoc. 2019 Mar 6;8(3):e11707. doi: 10.2196/11707 (PMC6442316; doi:10.2196/11707)
Supplement: Multimedia Appendix 1 [file resprot_v8i3e11707_app1.pdf]

Multimedia Appendix 1. Sample Size and Power calculations.

Power = 90%, α=0.05, $Z_{\alpha/2}$=1.96, Z_{β}=1.28, δ_B =4				
**Effect size (d=Δ/δ_B)**	**Δ (in GI units)**	**N/arm**	**Total N**	**N_{new} (30% Attrition)**
0.2	0.8	525	1050	1500
0.5	2.0	84	168	240
0.8	3.2	33	66	94
1.0	4.0	21	42	60

Power = 90%, α=0.05, $Z_{\alpha/2}$=1.96, Z_{β}=1.28, δ_B =5				
**Effect size (d=Δ/δ_B)**	**Δ (in GI units)**	**N/arm**	**Total N**	**N_{new} (30% Attrition)**
0.2	1.0	525	1050	1500
0.5	2.5	84	168	240
0.8	4.0	33	66	94
1.0	5.0	21	42	60

Power = 80%, α=0.05, $Z_{\alpha/2}$=1.96, Z_{β}=0.84, δ_B =4				
**Effect size (d=Δ/δ_B)**	**Δ (in GI units)**	**N/arm**	**Total N**	**N_{new} (30% Attrition)**
0.2	0.8	392	784	1120
0.5	2.0	63	126	180
0.8	3.2	25	50	71
1.0	4.0	16	32	46

Power = 80%, α=0.05, $Z_{\alpha/2}$=1.96, Z_{β}=0.84, δ_B =5				
**Effect size (d=Δ/δ_B)**	**Δ (in GI units)**	**N/arm**	**Total N**	**N_{new} (30% Attrition)**
0.2	1.0	392	784	1120
0.5	2.5	63	126	180
0.8	4.0	25	50	71
1.0	5.0	16	32	46

Formula for sample size calculation

$$N = \frac{2(Z_{\alpha/2} + Z_{\beta})^2\,\boldsymbol{\delta}^2}{\boldsymbol{\Delta}^2}$$

New sample size with attrition in mind:

$$N_{new} = \frac{n}{1-L}$$

Where **n** is the total number of subjects in each group not accounting for loss to follow-up and **L** is the loss to follow-up rate.
