# Supplementary material for: Healthy Eating and Active Living for Diabetes-Glycemic Index (HEALD-GI): Protocol for a Pragmatic Randomized Controlled Trial
Source: JMIR Res Protoc. 2019 Mar 6;8(3):e11707. doi: 10.2196/11707 (PMC6442316; doi:10.2196/11707)
Supplement: Multimedia Appendix 2 [file resprot_v8i3e11707_app2.pdf]

CFDR Scientific Review Committee Feedback
Steven Johnson

The following are comments on your proposal from the reviewers, some of which may be helpful in conducting the research.

The proposal is within the interests and objectives of CFDR. If the results show that intensive education about GI results in greater uptake and real dietary change, and improved glycemic control in T2DM, then it will be an important study for dietetics. It has the potential to change how dietitians counsel surrounding the GI. However, it is unclear if the intervention proposed in the study will have any influence on dietetic practice specifically—or simply improve adherence and adoption of GI by patients.

The literature demonstrated excellent knowledge of the field. Although it was mentioned, it was somewhat surprising that little credence was given to the Sacks et al. study in 2015 that suggested GI was of limited value in normal individuals. This needs to be considered in interpreting the results of the study.

The objectives of the project are achievable. The study is well designed to test the hypothesis.

The methodology appears to be appropriate and well outlined including thorough sample size justification. The approach to the statistical analysis is good. There are two reservations with regards to the statistical approach. First: Both the control and the test arm get the basic CDA + CFG literature; however, the test arm gets the added package. This additional attention alone may result in improved glycemic outcomes, unrelated necessarily to the specific effect of GI. Would similarly intense intervention regarding general dietary guidelines in the control group would be useful to rule out this possible confounder? Second, the group is using a simple randomization scheme. It is suggested to use random permuted blocks which will ensure better balance and avoid "runs" that would introduce imbalance into group size. This is especially the case when the study requires a small sample size only, an imbalance could throw off the statistical power.

Overall, the budget is well-defined and justified, although some concern was raised about spending $350 on envelopes.

The PI does not appear to have extensive experience with the GI concept, but does have extensive publications in the field of behavioural interventions for T2DM. No information is provided about team members and their expertise. The investigator appears to have appropriate support (e.g., RA) and infrastructure but this is not very clear.
